# Six stroma-based RNA markers diagnostic for prostate cancer in European-Americans validated at the RNA and protein levels in patients in China

**DOI:** 10.18632/oncotarget.4430

**Published:** 2015-06-19

**Authors:** Jianguo Zhu, Cong Pan, Jun Jiang, Mingsen Deng, Hengjun Gao, Bozhao Men, Michael McClelland, Dan Mercola, Wei-De Zhong, Zhenyu Jia

**Affiliations:** ^1^ Guizhou Provincial Key Laboratory of Computational Nano-Material Science, Guizhou Normal College, Guiyang, China; ^2^ Department of Urology, Guizhou Provincial People's Hospital, Guizhou, China; ^3^ Department of Chemical Physics, University of Science and Technology of China, Hefei, China; ^4^ National Engineering Center for Biochip at Shanghai, Shanghai, China; ^5^ Department of Microbiology and Molecular Genetics, University of California, Irvine, Irvine, CA, USA; ^6^ Department of Pathology and Laboratory Medicine, University of California, Irvine, Irvine, CA, USA; ^7^ Department of Urology, Guangdong Key Laboratory of Clinical Molecular Medicine and Diagnostics, Guangzhou First People's Hospital, Guangzhou Medical University, Guangzhou, China; ^8^ Department of Statistics, The University of Akron, Akron, OH, USA; ^9^ Department of Family and Community Medicine, Northeast Ohio Medical University, Rootstown, OH, USA

**Keywords:** prostate cancer, microenvironment, stroma, diagnosis, race

## Abstract

We previously analyzed human prostate tissue containing stroma near to tumor and from cancer-negative tissues of volunteers. Over 100 candidate gene expression differences were identified and used to develop a classifier that could detect nearby tumor with an accuracy of 97% (sensitivity = 98% and specificity = 88%) based on 364 independent test cases from primarily European American cases. These stroma-based gene signatures have the potential to identify cancer patients among those with negative biopsies. In this study, we used prostate tissues from Chinese cases to validate six of these markers (*CAV1, COL4A2, HSPB1, ITGB3, MAP1A and MCAM*). In validation by real-time PCR, four genes (*COL4A2*, *HSPB1*, *ITGB3*, and *MAP1A*) demonstrated significantly lower expression in tumor-adjacent stroma compared to normal stroma (*p* value ≤ 0.05). Next, we tested whether these expression differences could be extended to the protein level. In IHC assays, all six selected proteins showed lower expression in tumor-adjacent stroma compared to the normal stroma, of which COL4A2, HSPB1 and ITGB3 showed significant differences (*p* value ≤ 0.05). These results suggest that biomarkers for diagnosing prostate cancer based on tumor microenvironment may be applicable across multiple racial groups.

## INTRODUCTION

After years of intensive research, prostate cancer remains one of the major men's health issues worldwide and the second leading cause of cancer related death in males in United States. In China the disease was comparatively less frequent but the incidence is rapidly increasing to levels comparable to Europe and North America [1, 2].

Accurate diagnosis of the disease, especially the malignant type, is critical for optimal patient care. However, even the best current methods, including transrectal ultrasound (TRUS) procedures, may miss up to 30% of clinically significant prostate cancers who give false negative results on initial biopsy [3, 4]. These false negative biopsies contain ample stroma and other types of tissues (tumor microenvironment) that may be close to tumors. The tumor influences adjacent tissues through so-called paracrine mechanisms. It has been reported that the interaction and crosstalk between tumor and its microenvironment together contribute to tumor progression [5-12].

We previously developed a gene expression classifier which can diagnose the presence of prostate tumor using tumor adjacent stroma tissue. Almost exclusively European American (EA) cases in North America were used in that study [13]. We compared tumor-adjacent stroma to the stroma from normal subjects using fresh-frozen tissues and detected hundreds of gene expression differences. After correcting for age-related expression changes, we identified 131 genes that are reliably altered in RNA expression between tumor adjacent stroma and normal stroma. We then developed an expression classifier with these 131 genes using PAM [14]. The classifier was then tested on 364 independent cases with an accuracy of 97% (sensitivity = 98% and specificity = 88%) [13].

Racial and ethnic differences in outcome for prostate cancer (PCa) in the United States have been well documented [15-17]. The incidence of prostate cancer is lower in Asia [18]. These differences raise the question of whether the biomarkers for early diagnosis are also influenced by race. To determine if these diagnostic markers were broadly applicable for Chinese patients and, further, if they were applicable at the protein level, we selected six genes (*CAV1*, *COL4A2*, *HSPB1*, *ITGB3*, *MAP1A* and *MCAM*) from the initial 131 gene expression differences between tumor adjacent stroma and normal stroma in North American cases. Expression of RNA from the six genes was measured using real-time PCR with prostate samples from a collection of Chinese cases (set A). Then, antibodies directed at the proteins encoded by these six genes were employed for immunohistochemistry assays on tissue microarrays which consisted of tissue samples from another collection of Chinese prostate cancer patients and Chinese normal subjects (set B). The results from both assays based on Chinese samples highly correlated with the results of the previous study based on North American cases, and extended them to the protein level, indicating that it may be feasible to develop a diagnostic assay applicable to multiple races. Investigation of the function of these genes in depth (and genes that are closely related to these genes) my help uncover the biology of etiology of the disease.

## RESULTS

We have previously identified 131 genes that are significantly differentially expressed between tumor adjacent stroma and normal stroma using North American cases [13]. In the current study, we selected six representative genes for validation using Chinese cases. Previous literature indicated that these six genes, i.e., *CAV1* [19, 20], *COL4A2* [21], *HSPB1* [22-24], *ITGB3* [25], *MAP1A* [26] and *MCAM* [27], were linked to prostate cancer progression. Furthermore, pathway analysis using these six genes showed that they are highly significantly associated with important transcription factors, such as AP-1, AP-2, DPP4, AML1ETO fusion protein, p53, PBX2, PPAR-gamma, and STAT3.

**Figure 1:**
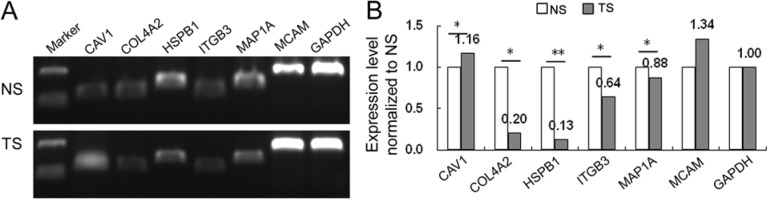
The results of RT-qPCR **A.** The electrophoresis of PCR product for six genes. **B.** Histogram for the relative expression levels for six genes. NS: normal stroma; TS: tumor adjacent stroma; *P < 0.05, **P < 0.01.

We used a set of Chinese cases from the tissue bank at Guangzhou First People's Hospital, China (Set A) for an RT-qPCR assay to test whether the RNA expression changes previously observed in primarily European-American patients, could be extended to Chinese patients. Stroma samples were collected from prostate cancer patients and normal prostate glands using LCM. RT-qPCR was used to measure the RNA expression changes of these six genes between tumor adjacent stroma and normal stroma. The results based on *t* test (Figure 1) indicate that *COL4A2*, *HSPB1*, *ITGB3*, and *MAP1A* were significantly less expressed in tumor adjacent stroma in comparison with normal stroma (*p* values < 0.05). The original images for the PCR agarose gel are given in Figure S1.

**Figure 2:**
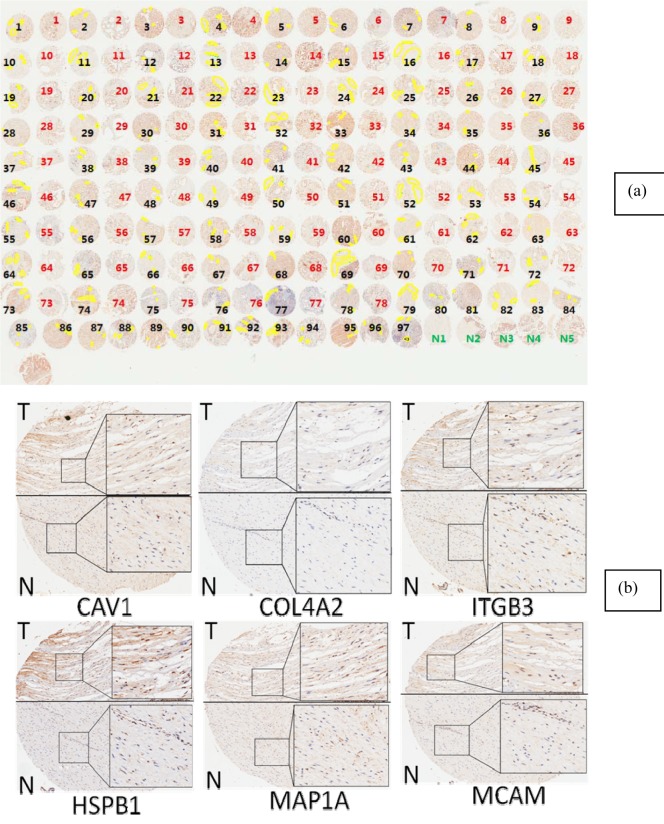
**a.** The stroma component selected for differential expression analysis. The images presented in the figure were obtained from IHC analysis of MCAM. Samples labeled with black/red represent, respectively, tumor-bearing and tumor-adjacent tissues from patients' prostate glands; samples labelled with green are prostate tissue from normal subjects. **b.** Representative figures obtained from IHC analysis of CAV1, COL4A2, ITGB3, HSPB1, MAP1A and MCAM. The samples labeled with T represent tumor-bearing/tumor-adjacent stroma area from patients' prostate glands; the samples labeled with N represent tumor-adjacent stroma (tumor free) area from the same patients' prostate glands.

We next used a Tissue Microarray to investigate whether these results could be extended, for the first time, to protein (Set B). TMAs were manually viewed by an experienced pathologist. From each tumor-bearing sample, we selected three stroma regions (marked yellow in Figure 2 (a)) representing the stroma that are adjacent to tumor epithelium regions. In our previous studies, we have learned that the field effect related to tumor paracrine mechanism depends on the tumor-stroma distance, for which the closer the stronger signal would be detected. Based on our experience, the optimal tumor-stroma distance for these markers is within 1mm [28-31]. So, we used pen tool in Aperio Imagescope system to select stroma that are close to tumor regions (Figure 2).

Enlarged pictures are shown in Figure 2 (b). For each of five normal tissues, three stroma regions were also selected for comparison (not shown in Figure 2 (a)). The selected areas were analyzed with the application “Positive Pixel Count V9”, by which the image data were translated to numerical data. The average intensity, which is the ratio of the sum of the intensities of positive signal (weak positive, positive and strong positive) and the sum of the number of positive signal (weak positive, positive and strong positive), is calculated and used for further analysis. For each of six proteins, the mean average intensities and standard deviations were calculated and compared between tumor adjacent stroma and normal stroma using the Student *t* test. The summary statistics based on the *t* test are presented in Table 1. All six proteins are less expressed in tumor adjacent stroma in comparison with the normal stroma. The differences are statistically significant for COL4A2, HSPB1 and ITGB3 (*p* values < 0.05), and the difference for MCAM showed the same trend (*p* value = 0.077, not significant but approaching significant level of 0.05).

**Table 1:** Summary statistics based on the *t* test

	Protein					
	CAV1	COL4A2	HSPB1	ITGB3	MAP1A	MCAM
MExp TS	195.3	188.9	177.7	190.3	188.6	194.4
Sd TS	5.3	10.4	12.1	6.4	5.9	7.6
MExp NS	197.4	195.7	183.4	196.0	190.0	197.2
Sd NS	6.6	4.5	8.2	3.3	5.7	5.3
*t* statistic	−1.18	−5.06	−2.52	−6.00	−0.89	−1.87
*P* value	0.255	3.26E-05	0.021	5.38E-06	0.388	0.077

MExp: mean expression; Sd: standard deviation

We determined if the protein expression levels for these six genes in tumor-adjacent stroma are correlated with clinical variables, such as age, Gleason scores, and stage, in order to explore the potential prognostic power for these biomarkers. There was no association between age and any of the six proteins, indicating the changes of these genes/proteins in tumor adjacent stroma are not due to aging, which is consistent with what has been reported in previous study based on American patient samples[13]. We found that only HSPB1 expression was weakly negatively associated with Gleason score (*P* value = 0.095) and tumor stage (*P* value = 0.136). Further validation is needed to prove this correlation. The protein encoded by HSPB1 translocates from the cytoplasm to the nucleus upon stress induction and is involved in stress resistance and actin organization [32].

## DISCUSSION

Stroma plays an important role in prostate carcinogenesis [33, 34]. It is also a key regulator in prostate function and cancer malignancy [35]. The stroma-epithelium crosstalk in prostate cancer has been intensively investigated in the literature [36]. Through paracrine mechanism, tumor epithelium cells send signals to adjacent stroma which arouses stroma to react by altering expression levels of involved genes/proteins, even if there is little morphological changes in stroma cells. These observations form the basis of continuing studies to identify biomarkers from stroma for disease diagnosis and prognosis [13, 32, 37].

Current diagnosis of presence of prostate tumor based on needle biopsies largely depends on examining the tissue for pathological epithelium. If biopsy samples do not contain recognizable tumor cells, which is very likely based on previous statistics presented [13], false negatives are unfortunately inevitable. However, if changes of genes/proteins in stroma due to the presence of tumor can be identified, instant companion test is possible to fill the void of early detection due to the inefficient biopsy procedure.

Previously, we identified 131 genes that are differentially expressed at the RNA level between tumor adjacent stroma and normal stroma in North American cases (Caucasians and African Americans) [13]. Here, we tested if some of these genes behave in a similar manner in Chinese patients, and extended the study beyond RNA expression to also include protein expression. In this study, we selected six genes, that had additionally been independently reported to be related to prostate cancer progression, *CAV1* [19, 20], *COL4A2* [21], *HSPB1* [22-24], *ITGB3* [25], *MAP1A* [26] and *MCAM* [27]. We tested these six genes using RT-qPCR based on another collection of 21 Chinese patient cases and 8 Chinese normal cases (Set A). Four genes (*COL4A2*, *HSPB1*, *ITGB3*, and *MAP1A*) were significantly less expressed in tumor adjacent stroma in comparison with normal stroma (*p* values < 0.05). These results are consistent with our RNA expression data, based on North American cases[13], indicating that it is possible to use these biomarkers to develop a diagnostic tool that is generic to various races.

By using a tissue microarray (TMA) of 97 tumor cores and 78 associated stroma, and 5 cores from normal donors, we showed that the proteins encoded by all these six genes were down-regulated in tumor-associated stroma of the prostate in Chinese cases (Set B), relative to normal stroma. Three proteins (COL4A2, HSPB1 and ITGB3) showed significant differences (*p* values < 0.05), and the difference for MCAM was weakly correlated (*p* value = 0.077).

The incidence of prostate cancer in the United States is significantly higher than in most other countries, particularly Asian countries, even though the incidence of histological lesions (abnormal pathology that is potentially precancerous) has been reported to be similar worldwide [35]. Environmental factors and region-specific diet have therefore been presumed to cause prostate carcinogenesis [36]. Our data indicated that at least one small subset of genes showed a similarly clinical signature between different biological populations. These expression markers have enormous potential for diagnosing the presence of tumor when biopsy samples do not contain recognizable tumors and may be the basis for a companion test that can help avoid unnecessary repeated prostate biopsies and reduce healthcare spending. The current study, aimed to extend this observation to Asian patients and from RNA to protein. These are proof-of-concept studies, and rigorous prospective trials are needed to confirm the potential application of these genes as diagnostic markers in clinical settings.

## MATERIALS AND METHODS

### Prostate tissues

The study was approved by the Institutional Review Board at Guizhou Normal College. Two collections of prostate tissue were used in this study, Set A for RT-qPCR validation and Set B for Tissue Microarray assay. The characteristics of all patient cases used in the current study are summarized in Table 2.

**Table 2:** Characteristics of patient cases

	Patent sets	
Characteristic	Set A	Set B
Number		
Patients	21	78
Normal subjects	8	5
Age for patients only		
Minimum	55	22
Maximum	87	90
Median	73	71
Gleason Score		
2-6	8 (38%)	24 (31%)
7 (3+4)	3 (14%)	19 (24%)
7 (4+3)	2 (10%)	17 (22%)
8-10	8 (38%)	17 (22%)
Insufficient data	0 (0%)	1 (1%)
Stage		
T1	5 (24%)	
T2	7 (33%)	60 (77%)
T3	9 (43%)	18 (23%)
Lymph Node Status		
N0		29 (37%)
N1		0 (37%)
Insufficient data		49 (63%)

The percentage in parenthesis represents the percent of patient cases (not including normal subjects) in each category.

Specimens used for RT-qPCR validation (Set A) include frozen samples (−80 °C) of 21 primary prostate cancer tissues (age median = 73, range 55-87) and eight normal prostate tissues (age median = 61.5, range 38-84), selected from the tissue bank at Guangzhou First People's Hospital, China. Using these samples was approved by the Research Ethics Committee of Guangzhou First People's Hospital, Guangzhou Medical University, China. Informed consent was obtained from all of the patients. All specimens were handled and made anonymous according to ethical and legal standards. None of the patients or subjects recruited for the study had chemotherapy or radiotherapy before the surgery. The prostate cancer tissues were collected from radical prostatectomy and TURP specimens, and the normal prostate tissues from cystoprostatectomy specimens for bladder cancer. Before RNA processing, all tissues were reconfirmed by HE staining and stroma tissues were collected from these samples using Laser Capture Micro-dissection.

Specimens used for Tissue Microarray assay (Set B) were obtained at the time of initial surgery. Prior consent from patients and approval from the Ethics Committees of Hospital was obtained for using these clinical materials for research purposes. All these specimens had confirmed pathological diagnosis and were classified according to the World Health Organization (WHO) criteria [38].

### RNA extraction and RT-qPCR

Stroma tissues were collected from 21 primary prostate cancer tissues and eight normal prostate tissues (Set A) using Laser Capture Micro-dissection. Total RNA of stroma tissues were extracted using RNAsimple Total RNA Kit (Cat No. DP419, TianGene, China) and measured by using UV spectrophotometer NanoDrop 2000 (Thermo Scientific, USA). RNA from 21 patient cases and eight normal cases were combined, respectively, and then analyzed using RT-qPCR to examine the mRNA expression levels of the genes of interest. The cDNA templates were synthesized from RNA samples by MMLV. The primer sequence information is given in Supplementary Table S1. Gene expression was determined using SYBR Green Real time PCR Master Mix (Cat No. QPK-201B, TOYOBO, Japan) and 0.2 μg of cDNA template. RT-qPCR was performed on a MyiQ.2 Two-Color RT-qPCR Detection System (Bio-Rad) following below amplification conditions: 5 min, 95°C; followed by 30 cycles of 10 seconds 95°C; 20 seconds 58°C; and 20 seconds 72°C. All assays were carried out by triplicate to control for technical variance. CT-values were determined using the IQ5 software (Bio-Rad). Gene expressions were normalized with *GAPDH* expression within each sample. Relative quantification of target gene expression was evaluated using the comparative cycle threshold (CT) method.

### Tissue microarray assay

Prostate Tissue Microarrays (TMA) were fabricated by Shanghai Outdo Biotech Co., Ltd (Cat. No. HPro-Ade180PG-02). On the TMAs, there are a total of 97 prostate cancer patient cases and five prostate glands from normal subjects (Set B). In addition, 78 out of 97 prostate tumor cases were paired as tumor-bearing tissues and adjacent tumor-free tissues from the same patient. All tissues were re-examined using a microscope by an experienced pathologist after transferred from a local hospital, based on which the pathological indices including Gleason score and stage were given to each patient.

Immunohistochemical staining of formalin-fixed and paraffin-embedded sections was performed using a standard immunohistochemistry (IHC) protocol. Briefly, after deparaffinization and rehydration using a Leica autostainer XL ST5010 system, the TMA slides were pretreated with 10mM sodium citrate buffer (pH 6.0) for 5-10 minutes in a microwave for antigen retrieval. The endogenous peroxidase was quenched by adding the hydrogen peroxide (3% H2O2 in 70% methanol) at room temperature for 15 minutes. After washing, the slides were blocked for 30 minutes. The blocking buffer was removed and the slides were then incubated for 1 hour with primary antibodies (CAV1, COL4A2, HSPB1, ITGB3, MAP1A and MCAM), respectively, with the optimized dilutions at room temperature. Slides were washed with the 1xPBS solution and further incubated with of DAKO Envision+/HRP for 30 minutes at room temperature. Detection was based on the use of the 3, 3′-diaminobenzidene as instructed (DAB kit, DAKO, Denmark). Slides were counterstained with hematoxylin before microscopic analysis. An H-Score was initially calculated based on scoring of stained cells according to published method [39].

### Image analysis

**Figure 3:**
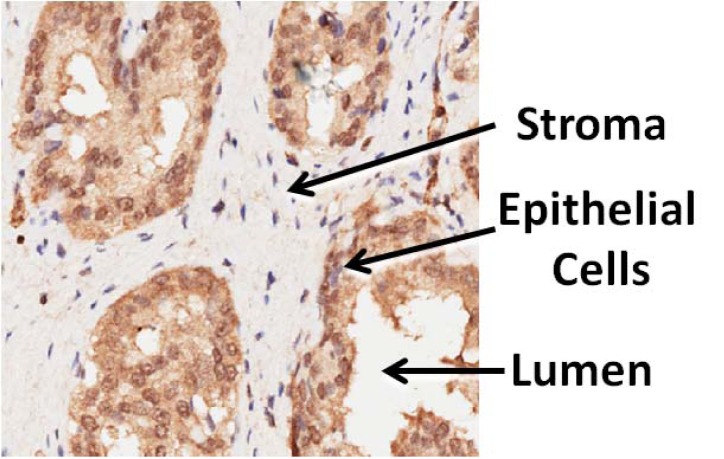
Schematic of an immunohistochemistry (IHC) stained prostate tissue microarray (TMA) cores showing heterogeneous compartment loci: lumen, epithelial cell, and stroma

The expressions of each protein in a TMA were measured by analyzing the staining signal intensity using Aperio image scope v11 (Aperio, USA). Briefly, in Aperio Imagescope windows. Epithelial cancer cells and stroma area were compartmentalized by an experienced pathologist using pen tool, based on typical pathological features (Figure 3). The brown staining (positive) in the intensely stained image and the blue staining (negative) in the least intensely stained area were selected for further data processing. The subsequent staining intensity was measured as the densitometry of the digital image (× 400), and the counted positive pixels were transformed to three intensity bins.

The application “Positive Pixel Count V9” of Aperio image scope v11 was used to select areas of interest (stroma component) from the each IHC image, and the image data were then translated to numerical data, such as intensities of positive signal, intensities of negative signal, number of positive, number of negative. The average intensity, which is the ratio of the sum of the intensities of positive signal (weak positive, positive and strong positive) and the sum of the number of positive signal (weak positive, positive and strong positive), is calculated and used for further statistical analysis.

### Statistical analysis

The Student *t* test was used to compare the staining intensities between tumor adjacent stroma and normal stroma in IHC assay, as well as the comparison of gene expression levels between tumor adjacent stroma and normal stroma in RT-qPCR assay. P value ≤ 0.05 was used for detecting significant difference in *t* tests.

### Gene ontology software

Using the Metacore software (GeneGo, Philadelphia, PA), an enrichment analysis was performed to identify significant biological pathways that resulted from our gene list. To limit false discovery and increase biological significance, pathways of interest had to meet the following conventional criteria, i.e., FDR < 5% and *p* < 0.05.

## SUPPLEMENTARY MATERIALS FIGURE AND TABLE



## References

[1] Hsing AW, Tsao L, Devesa SS (2000). International trends and patterns of prostate cancer incidence and mortality. Int J Cancer.

[2] Peng P, Gong YM, Bao PP, Ke JZ, Xiang YM, Zhang ML, Zheng Y (2012). Estimates and prediction of prostate cancer incidence, mortality and prevalence in China, 2008. Zhonghua liu xing bing xue za zhi = Zhonghua liuxingbingxue zazhi.

[3] Mian BM, Naya Y, Okihara K, Vakar-Lopez F, Troncoso P, Babaian RJ (2002). Predictors of cancer in repeat extended multisite prostate biopsy in men with previous negative extended multisite biopsy. Urology.

[4] Leite KR, Camara-Lopes LH, Cury J, Dall'oglio MF, Sanudo A, Srougi M (2008). Prostate cancer detection at rebiopsy after an initial benign diagnosis: results using sextant extended prostate biopsy. Clinics.

[5] Cunha GR, Hayward SW, Dahiya R, Foster BA (1996). Smooth muscle-epithelial interactions in normal and neoplastic prostatic development. Acta anatomica.

[6] Tlsty TD, Hein PW (2001). Know thy neighbor: stromal cells can contribute oncogenic signals. Current opinion in genetics & development.

[7] Cunha GR, Hayward SW, Wang YZ, Ricke WA (2003). Role of the stromal microenvironment in carcinogenesis of the prostate. Int J Cancer.

[8] Kiaris H, Chatzistamou I, Kalofoutis C, Koutselini H, Piperi C, Kalofoutis A (2004). Tumour-stroma interactions in carcinogenesis: basic aspects and perspectives. Molecular and cellular biochemistry.

[9] Cunha GR, Cooke PS, Kurita T (2004). Role of stromal-epithelial interactions in hormonal responses. Archives of histology and cytology.

[10] Tlsty TD, Coussens LM (2006). Tumor stroma and regulation of cancer development. Annual review of pathology.

[11] Rebbeck TR, Devesa SS, Chang BL, Bunker CH, Cheng I, Cooney K, Eeles R, Fernandez P, Giri VN, Gueye SM, Haiman CA, Henderson BE, Heyns CF, Hu JJ, Ingles SA, Isaacs W (2013). Global patterns of prostate cancer incidence, aggressiveness, and mortality in men of african descent. Prostate cancer.

[12] Hayward SW, Grossfeld GD, Tlsty TD, Cunha GR (1998). Genetic and epigenetic influences in prostatic carcinogenesis (review). Int J Oncol.

[13] Jia Z, Wang Y, Sawyers A, Yao H, Rahmatpanah F, Xia XQ, Xu Q, Pio R, Turan T, Koziol JA, Goodison S, Carpenter P, Wang-Rodriguez J, Simoneau A, Meyskens F, Sutton M (2011). Diagnosis of prostate cancer using differentially expressed genes in stroma. Cancer Res.

[14] Tibshirani R, Hastie T, Narasimhan B, Chu G (2002). Diagnosis of multiple cancer types by shrunken centroids of gene expression. Proc Natl Acad Sci U S A.

[15] Powell IJ (2007). Epidemiology and pathophysiology of prostate cancer in African-American men. The Journal of urology.

[16] DeSantis C, Naishadham D, Jemal A (2013). Cancer statistics for African Americans, 2013. CA: a cancer journal for clinicians.

[17] Hsing AW, Yeboah E, Biritwum R, Tettey Y, De Marzo AM, Adjei A, Netto GJ, Yu K, Li Y, Chokkalingam AP, Chu LW, Chia D, Partin A, Thompson IM, Quraishi SM, Niwa S (2014). High prevalence of screen detected prostate cancer in West Africans: implications for racial disparity of prostate cancer. The Journal of urology.

[18] Xia SJ, Cui D, Jiang Q (2012). An overview of prostate diseases and their characteristics specific to Asian men. Asian journal of andrology.

[19] Sotgia F, Martinez-Outschoorn UE, Pavlides S, Howell A, Pestell RG, Lisanti MP (2011). Understanding the Warburg effect and the prognostic value of stromal caveolin-1 as a marker of a lethal tumor microenvironment. Breast Cancer Res.

[20] Ayala G, Morello M, Frolov A, You S, Li R, Rosati F, Bartolucci G, Danza G, Adam RM, Thompson TC, Lisanti MP, Freeman MR, Di Vizio D (2013). Loss of caveolin-1 in prostate cancer stroma correlates with reduced relapse-free survival and is functionally relevant to tumour progression. The Journal of pathology.

[21] Gorlov IP, Byun J, Gorlova OY, Aparicio AM, Efstathiou E, Logothetis CJ (2009). Candidate pathways and genes for prostate cancer: a meta-analysis of gene expression data. BMC medical genomics.

[22] Cornford PA, Dodson AR, Parsons KF, Desmond AD, Woolfenden A, Fordham M, Neoptolemos JP, Ke Y, Foster CS (2000). Heat shock protein expression independently predicts clinical outcome in prostate cancer. Cancer Res.

[23] Shiota M, Bishop JL, Nip KM, Zardan A, Takeuchi A, Cordonnier T, Beraldi E, Bazov J, Fazli L, Chi K, Gleave M, Zoubeidi A (2013). Hsp27 regulates epithelial mesenchymal transition, metastasis, and circulating tumor cells in prostate cancer. Cancer Res.

[24] Vasiljevic N, Ahmad AS, Beesley C, Thorat MA, Fisher G, Berney DM, Moller H, Yu Y, Lu YJ, Cuzick J, Foster CS, Lorincz AT (2013). Association between DNA methylation of HSPB1 and death in low Gleason score prostate cancer. Prostate cancer and prostatic diseases.

[25] Xu L, Wang Z, Li XF, He X, Guan LL, Tuo JL, Wang Y, Luo Y, Zhong HL, Qiu SP, Cao KY (2013). Screening and identification of significant genes related to tumor metastasis and PSMA in prostate cancer using microarray analysis. Oncology reports.

[26] Yang B, Bhusari S, Kueck J, Weeratunga P, Wagner J, Leverson G, Huang W, Jarrard DF (2013). Methylation profiling defines an extensive field defect in histologically normal prostate tissues associated with prostate cancer. Neoplasia.

[27] Liu JW, Nagpal JK, Jeronimo C, Lee JE, Henrique R, Kim MS, Ostrow KL, Yamashita K, van Criekinge V, Wu G, Moon CS, Trink B, Sidransky D (2008). Hypermethylation of MCAM gene is associated with advanced tumor stage in prostate cancer. The Prostate.

[28] Lander AD, Nie Q, Wan FY (2005). Spatially distributed morphogen production and morphogen gradient formation. Mathematical biosciences and engineering : MBE.

[29] Lander AD, Nie Q, Wan FY (2007). Membrane-associated non-receptors and morphogen gradients. Bulletin of mathematical biology.

[30] Merkin JH, Sleeman BD (2005). On the spread of morphogens. Journal of mathematical biology.

[31] Zhang YT, Lander AD, Nie Q (2007). Computational analysis of BMP gradients in dorsal-ventral patterning of the zebrafish embryo. Journal of theoretical biology.

[32] Larkin SE, Holmes S, Cree IA, Walker T, Basketter V, Bickers B, Harris S, Garbis SD, Townsend PA, Aukim-Hastie C (2012). Identification of markers of prostate cancer progression using candidate gene expression. British journal of cancer.

[33] Tuxhorn JA, Ayala GE, Rowley DR (2001). Reactive stroma in prostate cancer progression. The Journal of urology.

[34] Cunha GR, Hayward SW, Wang YZ (2002). Role of stroma in carcinogenesis of the prostate. Differentiation; research in biological diversity.

[35] Hagglof C, Bergh A (2012). The stroma-a key regulator in prostate function and malignancy. Cancers.

[36] Niu YN, Xia SJ (2009). Stroma-epithelium crosstalk in prostate cancer. Asian journal of andrology.

[37] Jia Z, Rahmatpanah FB, Chen X, Lernhardt W, Wang Y, Xia XQ, Sawyers A, Sutton M, McClelland M, Mercola D (2012). Expression changes in the stroma of prostate cancer predict subsequent relapse. PloS one.

[38] Epstein JI, Allsbrook WC, Amin MB, Egevad LL, Committee IG (2005). The 2005 International Society of Urological Pathology (ISUP) Consensus Conference on Gleason Grading of Prostatic Carcinoma. The American journal of surgical pathology.

[39] Ishibashi H, Suzuki T, Suzuki S, Moriya T, Kaneko C, Takizawa T, Sunamori M, Handa M, Kondo T, Sasano H (2003). Sex steroid hormone receptors in human thymoma. The Journal of clinical endocrinology and metabolism.

