# Supplementary material for: Six stroma-based RNA markers diagnostic for prostate cancer in European-Americans validated at the RNA and protein levels in patients in China
Source: Oncotarget. 2015 Jun 19;6(18):16757–65. doi: 10.18632/oncotarget.4430 (PMC4599305; doi:10.18632/oncotarget.4430)
Supplement: Supplementary file 1 [file oncotarget-06-16757-s001.pdf]

Six stroma-based RNA markers diagnostic for prostate cancer in European-Americans validated at the RNA and protein levels in patients in China

Supplementary Material

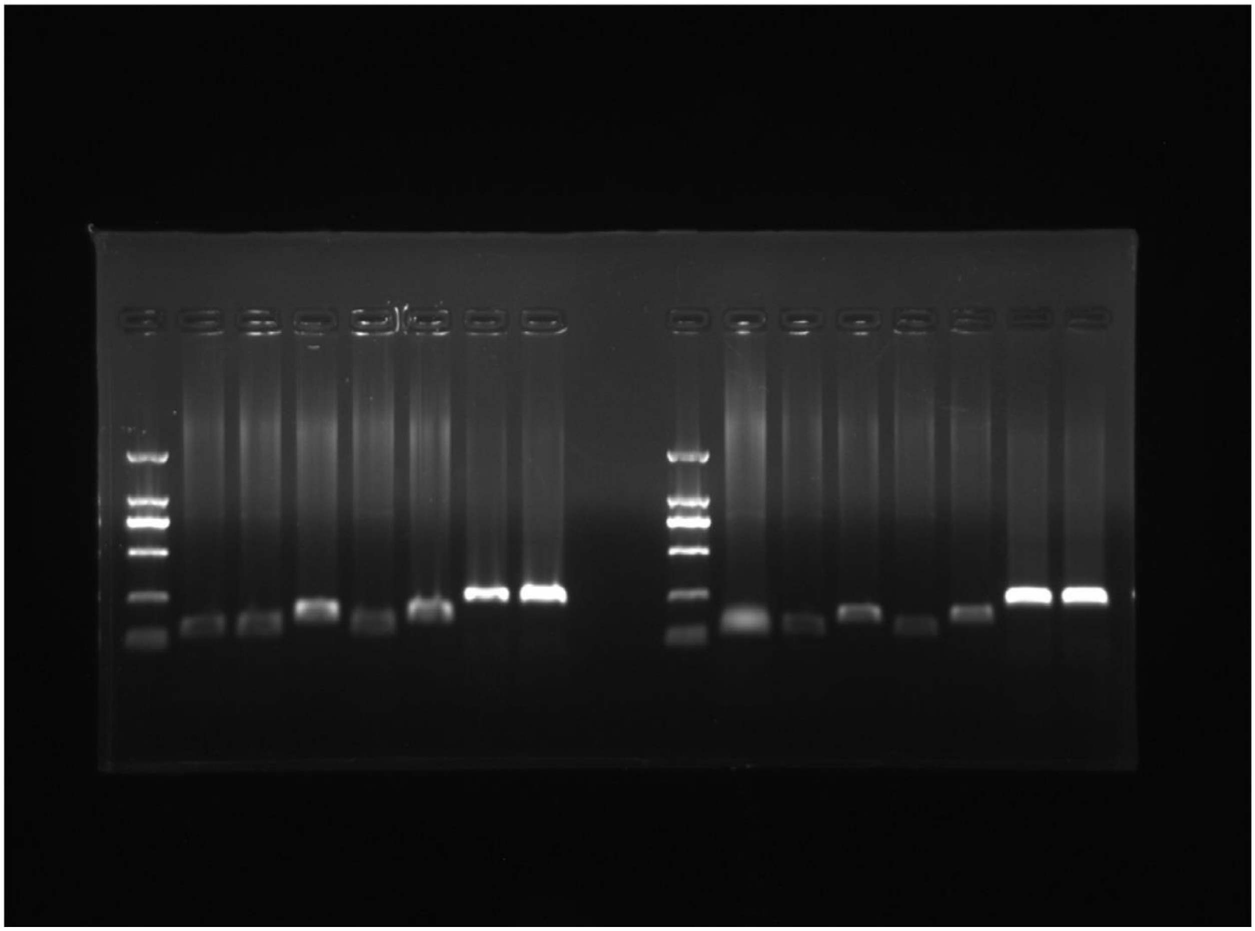

Figure S1: Original images for the PCR agarose gel. Left panel is normal stroma (NS) and right panel is Tumor adjacent stroma (TS).

Table S1: Primer List for the six selected genes.

Gene	Direction	Primer	PCR production length
CAV1	F	CAGTGCATCAGCCGTGTCTA	103
	R	TCTGCAAGTTGATGCGGACA	
COL4A2	F	CATCACACCCCCTTCCAACA	80
	R	CCCGATAACCTTTCAGGCCA	
HSPB1	F	AGCTGACGGTCAAGACCAAG	224
	R	TGGGATGGTGATCTCGTTGG	
ITGB3	F	CCCAGATGCCTGCACCTTTA	110
	R	ACTCAATCTCGTCACGGCAG	
MAP1A	F	GAGAGGCTTCCTCCTACACC	186
	R	GTCTCCCCCAGGCTCTAAAC	
MCAM	F	GAAGCATGGGGCTTCCCAG	234
	R	GCACACGGAAGATGAGCGTC	
GAPDH	F	CATGGGTGTGAACCATGAGAAGTA	239
	R	CAGTAGAGGCAGGGATGATGTTCT	
